# Temporal genetic changes in human bocavirus 1 in Fukushima, Japan, from 2018 to 2024

**DOI:** 10.1099/jmm.0.002165

**Published:** 2026-05-12

**Authors:** Tatsuya Shirai, Miyuki Kawase, Satoko Sugimoto, Masatoshi Kakizaki, Takashi Okura, Yohei Kume, Hisao Okabe, Sakurako Norito, Hiroko Sakuma, Shigeo Suzuki, Hayato Go, Mitsuaki Hosoya, Koichi Hashimoto, Kazuya Shirato

**Affiliations:** 1 Department of Respiratory Viruses, National Institute of Infectious Diseases, Japan Institute for Health Security, 4-7-1 Gakuen, Musashimurayama, Tokyo 208-0011, Japan; 2 Department of Respiratory Medicine, Faculty of Medicine, Kyorin University, Mitaka, Tokyo 181-8611, Japan; 3 Research Center for Biosafety, Laboratory Animal and Pathogen Bank, National Institute of Infectious Diseases, 4-7-1 Gakuen, Musashimurayama, Tokyo 208-0011, Japan; 4 Department of Pediatrics, School of Medicine, Fukushima Medical University, 1st Hikarigaoka, Fukushima 960-1295, Japan; 5 Hoshi General Hospital, 159-1 Mukaigawara, Koriyama, Fukushima 963-8501, Japan; 6 Ohara General Hospital, 6-1 Uwamachi, Fukushima 960-8611, Japan; 7 Department of Perinatology and Pediatrics for Regional Medical Support, Fukushima Medical University, Fukushima 960-1295, Japan

**Keywords:** genotype, human bocavirus 1, phylogenetic analysis

## Abstract

**Introduction.** Human bocavirus 1 (HBoV1) is a member of *Bocaparvovirus primate1* and is associated with paediatric respiratory infections. Although genetic grouping has been described, it remains unclear whether temporal genetic changes occur among clinically circulating HBoV1 strains.

**Hypothesis/Gap Statement.** HBoV1 shows limited genetic diversity, but temporal genetic changes may occur in circulating strains.

**Aim.** To investigate whether temporal genetic changes are present in HBoV1 isolates collected in Fukushima, Japan, between 2018 and 2024.

**Methodology.** Nasopharyngeal swabs were collected from paediatric inpatients with acute respiratory infections. Thirty-two HBoV1 isolates were obtained using air–liquid interface cultures of human bronchial/tracheal epithelial cells. Nearly complete genomes were sequenced by next-generation sequencing and analysed together with additional reference sequences (total *n*=100, including outgroups) using phylogenetic and time-scaled methods.

**Results.** Phylogenetic analyses classified the 32 Fukushima isolates into Group 1 (*n*=12) and Group 2 (*n*=20). Group 2 predominated in 2018, Group 1 from 2019 to 2021 and Group 2 again from 2022 to 2024, indicating temporal shifts in circulating strains. The average number of nucleotide differences among HBoV1 genomes was 13.9 (maximum 55), with Groups 1 and 2 diverging around 2004–2005. Divergence was concentrated in the VP genes, and synonymous substitutions within VP genes overlapped the ORFx reading frame, leading to distinct amino acid differences in ORFx.

**Conclusion.** Clinically circulating HBoV1 undergoes temporal genetic change, with group differentiation determined primarily by VP genes and uniquely by ORFx at the amino acid level. These findings suggest a potential biological role for ORFx in the HBoV1 life cycle. Understanding these temporal genetic shifts is important for enhancing the accuracy of molecular surveillance tools, particularly for distinguishing genetically diverse HBoV1 strains.

## Introduction

*Bocaparvovirus primate1* belongs to the genus *Bocaparvovirus*, subfamily *Parvovirinae* and family *Parvoviridae* and includes human bocavirus 1 (HBoV1) and HBoV3. In contrast, HBoV2 and HBoV4 are classified as members of *Bocaparvovirus primate2* [1]. The HBoV genome is ~5.5 kb in length, composed of single-stranded, heterotelomeric DNA, and contains three major ORFs that encode non-structural proteins (NSs), a nuclear phosphoprotein 1 (NP1) and viral capsid proteins (VPs) [2].

HBoV1 was first identified in Sweden in 2005 [3] and has since been associated with lower respiratory tract disease, both in co-infections and as a sole pathogen, underscoring its clinical relevance [4,6]. We previously reported that HBoV1 is frequently detected in specimens collected from paediatric inpatients with respiratory symptoms in Fukushima, Japan, with circulation predominantly from spring to summer [7,8]. During the coronavirus disease 2019 (COVID-19) pandemic, the incidence of enveloped viruses – readily inactivated by alcohol-based disinfectants – declined markedly due to enhanced hygiene measures. In contrast, non-enveloped viruses, including HBoV1, continued to be detected at levels similar to those observed before the pandemic [7,8].

Phylogenetic analyses have suggested that HBoV1 can be divided into three or four genetic groups, with one group typically predominating in a given year [9,10]. Although these phylogenetic groupings are widely recognized, previous studies have not identified consistent nucleotide or amino acid signatures that define each group. Reported differences in the VP region vary between studies and countries, and no specific mutation has been established as a reliable marker for group classification. Thus, the biological or molecular basis underlying these groupings remains unclear. In our previous studies, HBoV1 was consistently detected before, during and after the COVID-19 pandemic; however, temporal genetic trends have remained unclear. In this study, we isolated HBoV1 using air–liquid interface (ALI) cultures of human bronchial/tracheal epithelial cells (HBTECs) from paediatric specimens collected in Fukushima, Japan, between 2018 and 2024. Nearly complete viral genomes were sequenced using next-generation sequencing (NGS), and temporal genetic changes were analysed. However, it remains unknown whether HBoV1 undergoes measurable temporal genetic change during its circulation in human populations. We therefore hypothesized that genetic shifts occur over time in circulating HBoV1 lineages and aimed to identify such temporal changes by analysing nearly complete genomes isolated in Fukushima between 2018 and 2024.

## Methods

### Clinical specimens

Nasopharyngeal swabs were collected from paediatric inpatients with severe acute respiratory infections in Fukushima Prefecture between 2018 and 2024 [7,8].

### Virus isolation

Nucleic acids were extracted using either the QIAamp 96 Virus QIAcube HT Kit (Qiagen, Hilden, Germany) or the QIAamp Viral RNA Mini Kit (Qiagen), according to the manufacturer’s instructions. Elution with the QIAamp 96 Virus QIAcube HT Kit was performed by centrifugation. Although these kits are primarily designed for RNA, they also efficiently co-purify viral DNA because they do not include a DNase digestion step. This property has been widely utilized in previous studies detecting human bocavirus, as well as other DNA respiratory viruses, and these kits are routinely used for the extraction of both DNA and RNA respiratory viruses [7,11,13]. Respiratory viruses were detected by real-time reverse transcription-PCR (RT-PCR) using LightCycler instruments (Roche, Basel, Switzerland), as previously described [11,14]. HBoV1-positive specimens were subjected to virus isolation using HBTEC-ALI cultures [12,15, 16]. Briefly, HBTEC cells (FC-0035, LIFELINE Cell Technology, Frederick, MD, USA) were seeded onto 6.5 mm Transwell inserts (3470, Corning, One Riverfront Plaza, NY, USA). On the following day, the apical medium was removed, and the basal medium was replaced with differentiation medium. Cultures were maintained under ALI conditions at 37 °C in a humidified 5% CO₂ atmosphere for 4 weeks, with medium changes every 2–4 days, until well-differentiated polarized epithelial layers formed. For virus inoculation, 20 µl of clinical specimen or virus stock diluted 1 : 1 in 1% FCS-supplemented Dulbecco”s modified Eagle medium (DMEM) containing antibiotics (penicillin–streptomycin, gentamicin and fungizone) was applied to the apical surface of ALI cultures. After overnight incubation at 34 °C, cultures were washed four times with 1% FCS-DMEM. The fourth wash was collected as the day 0 (baseline) sample. To prevent mycoplasma contamination, the quinolone antimicrobial agent MC-210 (KAC Co., Ltd., Hyogo, Japan) was continuously added to the basolateral medium. Subsequent apical washes were performed on days 4, 7 and 11 and at weekly intervals, stored at −80 °C and analysed by real-time RT-PCR based on crossing point values [11,14].

### Sequencing analysis

NGS libraries were prepared with the NEBNext Ultra II RNA Library Prep Kit (New England Biolabs, Ipswich, MA, USA) and sequenced in 2×150 bp paired-end format on the DNBSEQ-G400 platform at AZENTA/GENEWIZ (Chelmsford, MA, USA). Although HBoV1 is a DNA virus, this workflow has been used for sequencing HBoV1 because it efficiently converts viral ssDNA into double-stranded cDNA suitable for Illumina-compatible library preparation [12,13]. In accordance with these previous studies, this kit yielded high-quality libraries with sufficient depth for nearly complete genome reconstruction of HBoV1. Raw reads were adapter-trimmed and assembled in CLC Genomics Workbench (versions 21.0.4–24.0.1; Qiagen) using *de novo* assembly and reference mapping to a representative HBoV1 genome (JQ923422.1). Consensus sequences were verified by alignment and manual inspection. Sequences were further analysed with Sequencher (version 5.4.6, Gene Codes Corporation, Ann Arbor, MI, USA). The median sequencing coverage of the 32 HBoV1 genomes was 7,316× (range, 15.7× to 151,635×). Statistical analyses were performed using SigmaPlot (version 14.5, Systat Software Inc., San Jose, CA, USA). Differences between groups were assessed using one-way ANOVA on ranks with Dunn’s test, with *P*<0.05 considered statistically significant.

### Phylogenetic analysis

A total of 32 HBoV1 sequences obtained in Fukushima were analysed together with additional nearly complete HBoV1 sequences (>5,000 nucleotides) retrieved from NCBI Virus (TaxID 689403). After removing sequences with ambiguous nucleotides (e.g. ‘N’) or without collection year information, 68 public sequences remained. These were combined with the 32 Fukushima isolates, resulting in a final dataset of 100 sequences used for phylogenetic and time-scaled analyses. Sequence alignment was performed using MAFFT (https://mafft.cbrc.jp/alignment/server/). Phylogenetic trees were constructed in mega (version 12.0.11), with sequences trimmed to nucleotide positions 102–5303 of reference JQ923422.1 for maximum comparability. The maximum likelihood method with 1,000 bootstrap replicates and a 70% site coverage cutoff (partial deletion) was used. Divergence times were estimated using the RelTime with Dated Tips (RTDT) method in mega [17].

## Results

### Genetic trends of HBoV1 in Fukushima, Japan

**Fig. 1.**
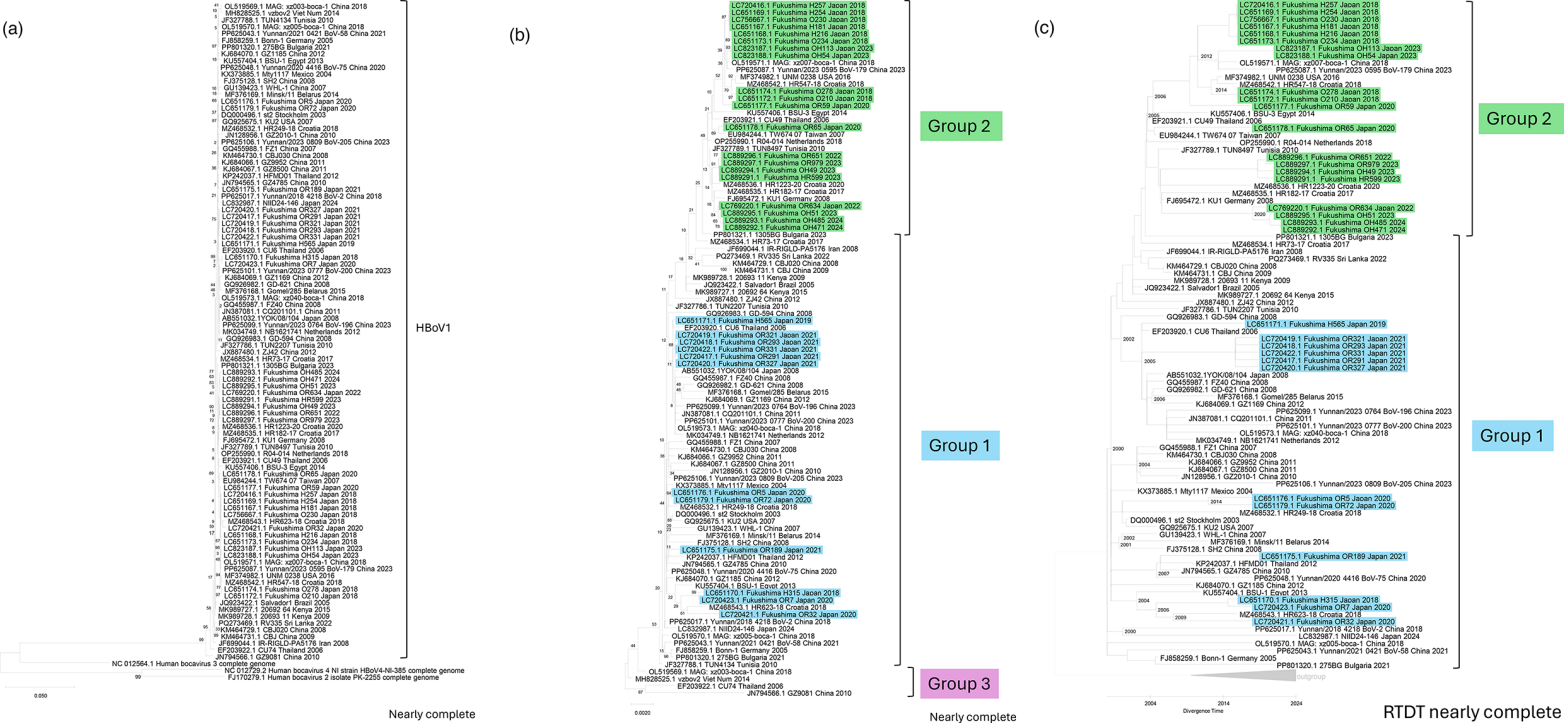
Phylogenetic trees of HBoV1 sequences. (**a**) Phylogenetic tree constructed in mega (version 12.0.11) using 97 HBoV1 sequences and 3 outgroup sequences (HBoV2–4), generated with the maximum likelihood method, 1,000 bootstrap replicates and a 70% site coverage cutoff (partial deletion). (**b**) Phylogenetic tree of HBoV1 sequences excluding outgroups. Sequences highlighted in light blue represent Group 1 Fukushima isolates, and those in light green represent Group 2 Fukushima isolates. All non-Fukushima sequences are shown in standard black font. (**c**) Time-scaled phylogenetic tree of nearly complete HBoV1 sequences with known collection years. The tree was constructed in mega (version 12.0.11) using the RTDT method, with Group 3 sequences as the outgroup, and generated with the maximum likelihood method, 1,000 bootstrap replicates and a 70% site coverage cutoff (partial deletion). Fukushima Group 1 isolates are highlighted in light blue, and Fukushima Group 2 isolates in light green.

Sequence similarity among HBoV1 genomes was very high (Fig. 1a). Genetic diversity became apparent only after excluding outgroups (Fig. 1b). The average number of nucleotide differences was 13.9 (range: 2–55) relative to the consensus sequence. Based on nearly complete genomic sequences, HBoV1 could be classified into three groups, consistent with a previous Japanese study [9]. Among the 32 Fukushima isolates collected between 2018 and 2024, 12 belonged to Group 1 and 20 to Group 2. The yearly distribution is summarized in Table 1. In 2018, most sequences belonged to Group 2. Group 1 predominated from 2019 to 2021, although only one sequence was available in 2019. Group 2 reappeared in 2022 and continued to be detected through 2024.

**Table 1.** Number of HBoV1 sequences in each group by year

	2018	2019	2020	2021	2022	2023	2024
Number of specimens tested	16	13	10	13	4	15	4
Number of sequences obtained	9	1	6	6	2	6	2
Group 1 sequences	1	1	4	6	0	0	0
Group 2 sequences	8	0	2	0	2	6	2

These year-to-year changes in predominant groups have not been previously documented and indicate temporal genetic shifts among circulating HBoV1 strains, at least in Japan. Using the RTDT method [17], divergence times were estimated based on collection years. The analysis indicated that Groups 1 and 2 diverged around 2004–2005 (Fig. 1c).

### Determinant of genetic trend of HBoV1

Although overall sequence diversity among HBoV1 genomes was low (maximum 55 nucleotides), the differences were concentrated in the VP genes. These genes encode three structural proteins, VP1, VP2 and VP3, which assemble into the viral capsid and are known to be immunogenic [18,19]. Group 1 sequences contained fewer nucleotide and amino acid differences relative to the consensus sequence, whereas Groups 2 and 3 exhibited statistically significant differences. In contrast, no significant variation was observed in the non-structural proteins (NS1 and NP1), suggesting that the structural proteins, particularly the VP genes, are the primary determinants of group differentiation.

**Fig. 2.**
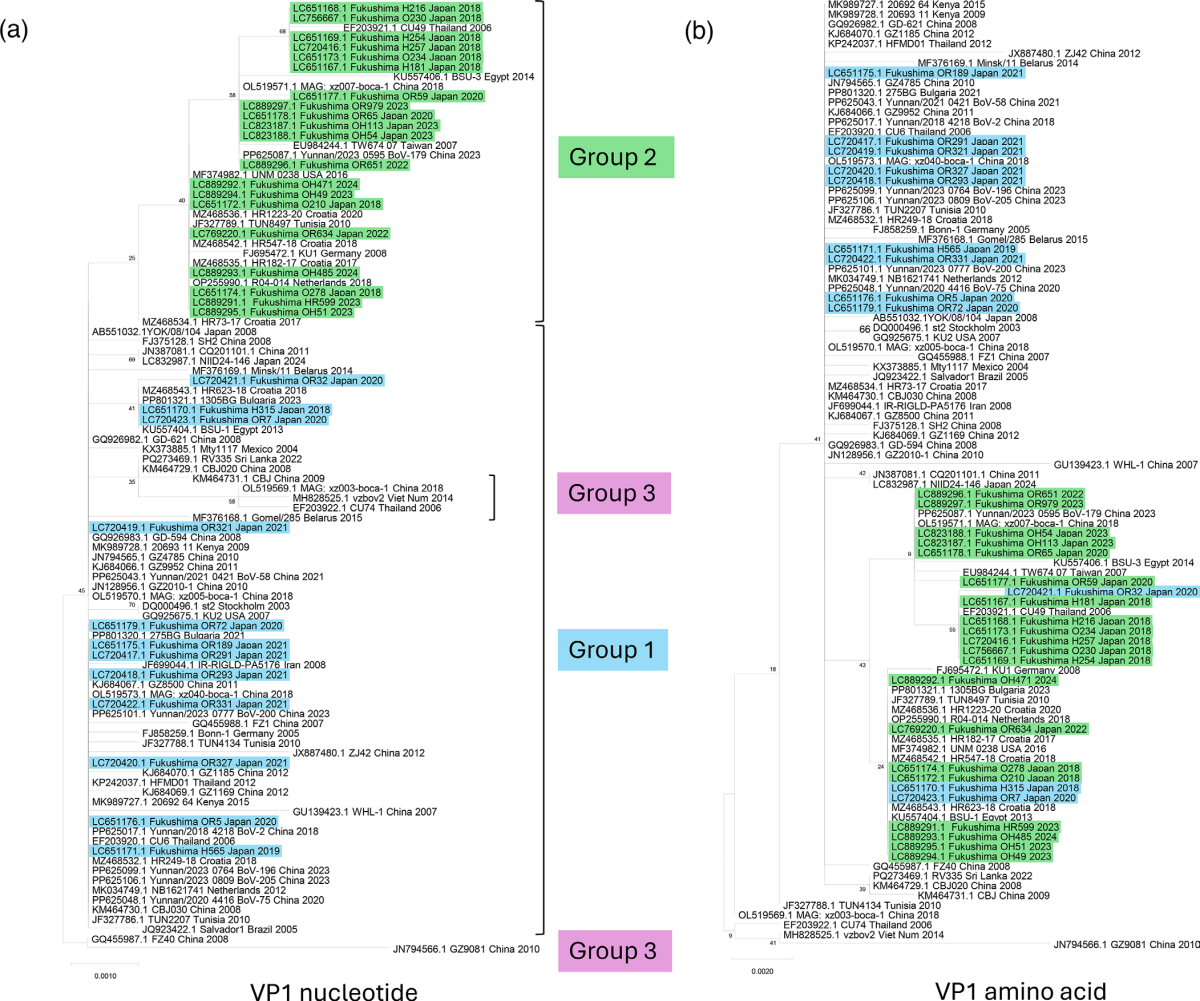

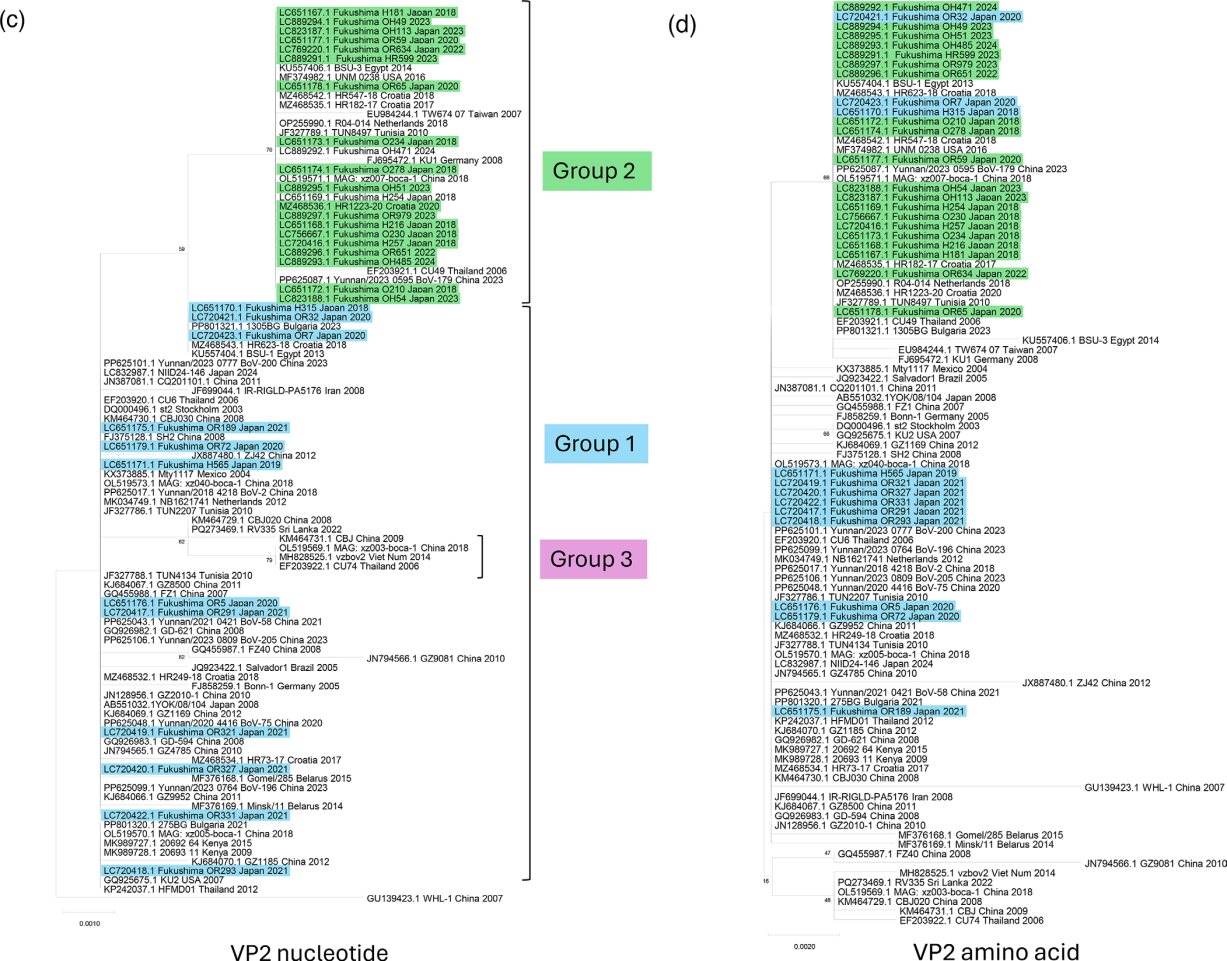

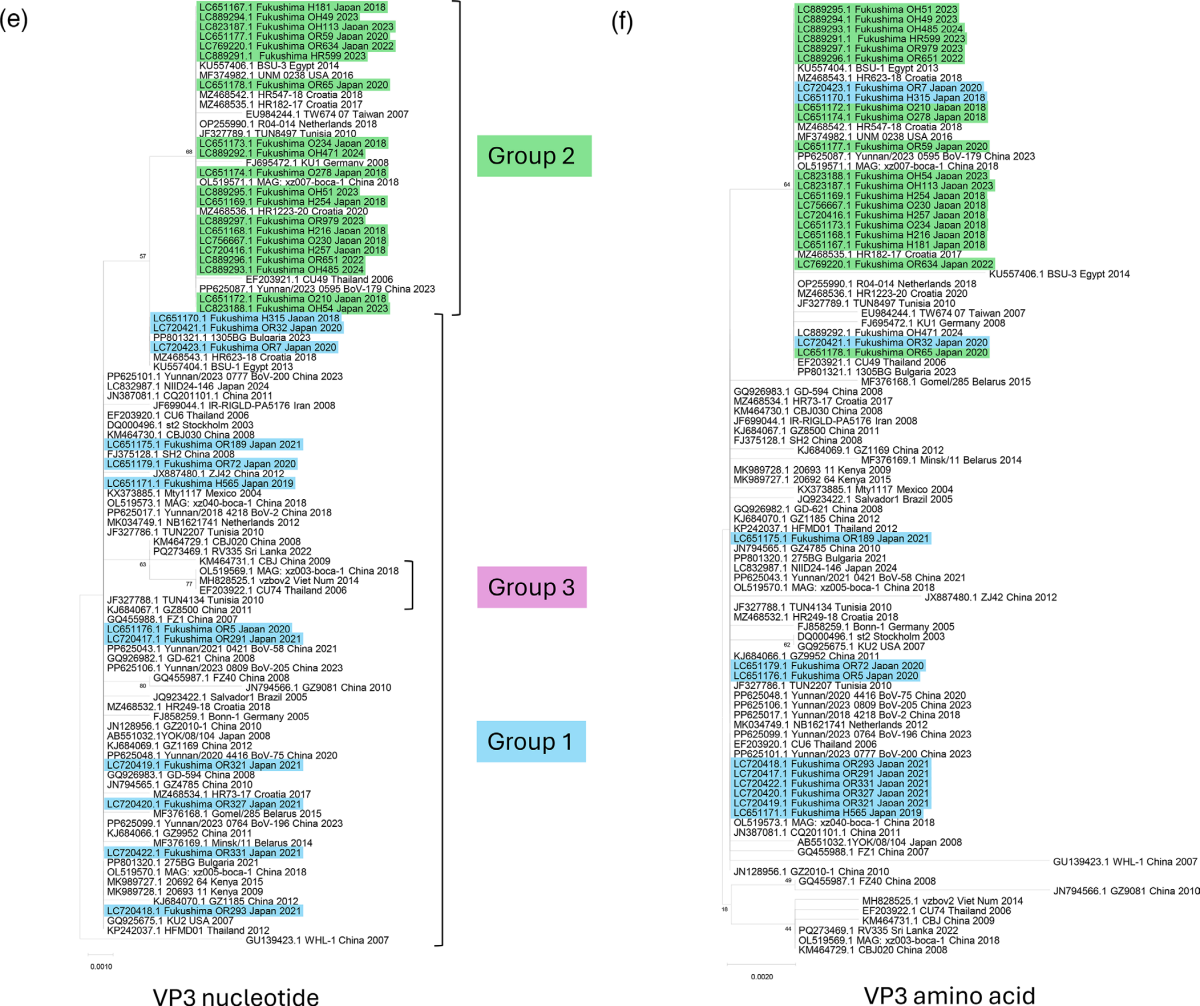

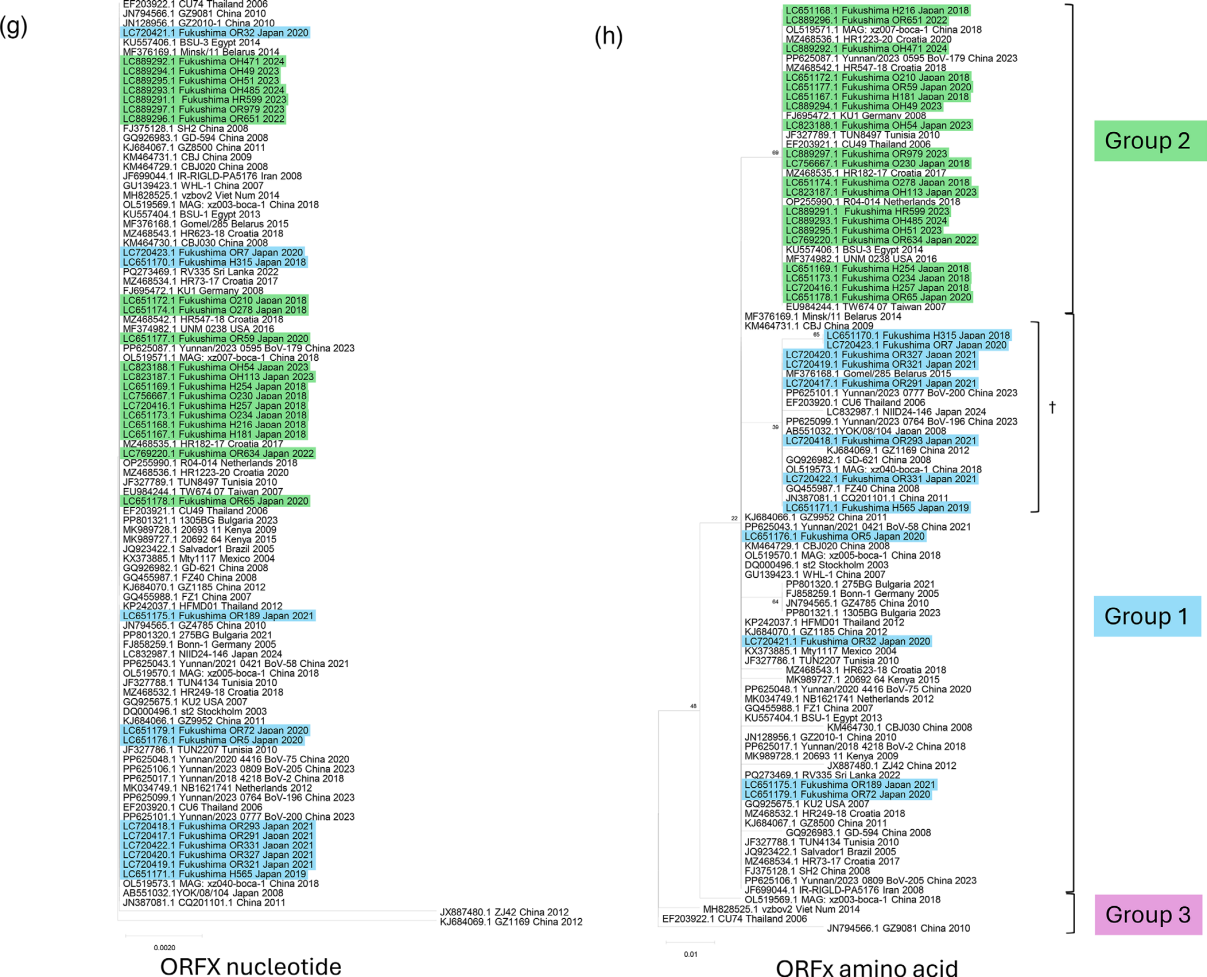
Phylogenetic trees of VP1–3 and ORFx sequences of HBoV1. Phylogenetic trees were constructed in mega (version 12.0.11) using 97 HBoV1 VP1 (a, nucleotide; b, amino acid), VP2 (c, nucleotide; d, amino acid), VP3 (e, nucleotide; f, amino acid) and ORFx (g, nucleotide; h, amino acid) sequences, with the maximum likelihood method, 1,000 bootstrap replicates and a 70% site coverage cutoff (partial deletion). Fukushima Group 1 isolates are highlighted in light blue, and Fukushima Group 2 isolates in light green. All other sequences are displayed in standard black font. Group labels are shown only in panels where the phylogenetic structure clearly supports distinct clustering of Groups 1 and 2. In panels b, d, f and g, the groups do not form monophyletic clades; therefore, group labels were intentionally omitted to avoid implying unsupported boundaries. ^†^, sub-cluster in Group 1 sequences.

Phylogenetic analysis of VP nucleotide sequences clearly separated Groups 1 and 2 (Fig. 2a, c and e), while Group 3 clustered within Group 1. VP amino acid phylogenies also distinguished the groups, although some Group 1 sequences – including three Fukushima isolates (H315, OR7 and OR32) – clustered within the Group 2 branch (Fig. 2b, d and f). No consistent amino acid substitutions were identified in the VP proteins between Groups 1 and 2; only a few scattered substitutions were observed.

Several VP1 amino acid substitutions have previously been proposed as potential lineage markers, including L40S, G96E and N474S [10]. However, these mutations did not correspond to group assignments in our dataset: L40S occurred in both Group 1 and Group 3, G96E was not detected, and N474S was present in nearly all sequences irrespective of group. These observations indicate that VP proteins do not encode reliable group-defining signatures.

Schildgen *et al*. reported a putative additional protein encoded by the ORFx region within the VP gene [20]. Although substitutions in ORFx were few, Groups 2 and 3 carried significantly more than Group 1 (Table 2). ORFx nucleotide sequences were highly conserved (Fig. 2g), whereas amino acid sequences clearly separated the groups (Fig. 2h). Group 2 consistently encoded isoleucine at position 111, whereas Group 1 encoded threonine. Group 3 also encoded threonine at position 111 but carried two to five additional substitutions. Within Group 1, a distinct sub-cluster encoded isoleucine at position 25 instead of threonine (see Fig. S1, available in the online Supplementary Material, for amino acid alignment). These data indicate that amino acid variation in ORFx is associated with group differentiation of HBoV1 genomes.

**Table 2.** Differences of nucleotides and amino acids among HBoV1 sequences

Nucleotide		All sequences	Group		
			1	2	3
Average number of differences (Min–Max)	Nearly complete	13.9 (2–55)	10.0 (2–24)	19.3 (14–29)*	31.0 (13–55)*
	VP1	9.0 (2–37)	5.2 (2–14)	14.8 (12–21)*	22.5 (12–37)*
	VP2	8.6 (2–35)	5.0 (2–13)	13.9 (12–20)*	21.3 (11–35)*
	VP3	8.5 (1–35)	4.9 (1–13)	13.8 (12–20)*	21.3 (11–35)*
	NS1	3.2 (0–13)	3.3 (0–13)	3.2 (0–10)	2.8 (1–5)
	NP1	1.1 (0–4)	1.2 (0–4)	0.8 (0–2)	2.0 (1–3)
	ORFx	1.6 (0–6)	0.7 (0–3)	3.0 (3–4)*	4.75 (4–6)*
**Amino acid**		**All sequences**	**Group**		
			**1**	**2**	**3**
Average number of differences (Min–Max)	VP1	1.21 (0–7)	0.66 (0–5)	1.94 (1–5)*	3.75 (2–7)*
	VP2	0.85 (0–5)	0.53 (0–5)	1.16 (1–4)*	2.50 (1–5)*
	VP3	0.81 (0–5)	0.53 (0–5)	1.16 (1–4)*	2.50 (1–5)*
	NS1	0.9 (0–11)	1.16 (0–11)	0.38 (0–2)	1.00 (0–2)
	NP1	0.21 (0–2)	0.30 (0–2)	0	0.50 (0–1)
	ORFx	0.83 (0–6)	0.57 (0–3)	1.00 (1)*	3.25 (2-6)*

* *P*<0.01, statistically significant relative to Group 1.

Importantly, the association between specific ORFx amino acid substitutions and HBoV1 group classification has not been previously reported. This represents the first evidence that ORFx, rather than VP amino acid sequences, provides the most reliable genotypic marker distinguishing HBoV1 lineages. Together, these findings demonstrate a genotype–group relationship in which synonymous VP substitutions affecting the overlapping ORFx frame serve as key molecular determinants of group differentiation.

## Discussion

This study demonstrated that HBoV1 genomes comprise several genetic groups and that temporal genetic changes occur in clinically circulating viruses. Group classification was determined primarily by VP genes, with additional involvement of the ORFx region. These findings provide new insights into the molecular evolution of HBoV1 and suggest that genetic diversification occurs during its circulation in human populations.

Shackelton *et al.* reported in 2005 that the rate of nucleotide substitution in ssDNA viruses is closer to that of RNA viruses than to dsDNA viruses [21]. Consistent with this, HBoV1 has been considered to evolve relatively rapidly, similar to other parvoviruses [22]. Bayesian analyses have estimated that HBoV1 diverged from its most recent common ancestor ~60–80 years ago, whereas HBoV4 diverged 200–300 years ago, with substitution rates of 8.6–9.0×10⁻⁴ substitutions/site/year [22,23]. In contrast, we observed only 13.9 nucleotide differences on average (maximum 55) among HBoV1 genomes. This lower-than-expected divergence likely reflects both the time-dependent rate phenomenon and strong purifying selection acting on the compact bocaparvovirus genome [22]. The average number of nucleotide differences was 10.0 in Group 1 and 19.3 in Group 2. Since our analysis indicated that Groups 1 and 2 diverged around 2004–2005, this suggests that fewer than 10 nucleotide substitutions have accumulated over the past two decades. These findings support the notion that the genetic evolution of HBoV1 is slower than would be expected from short-term substitution rate estimates.

A central finding of this study is that temporal genetic change in circulating HBoV1 is determined primarily by the VP region. The VP genes encode three structural proteins, VP1, VP2 and VP3, which form the viral capsid and are highly immunogenic; in particular, VP2 induces strong humoral and cellular immune responses [18]. It is therefore plausible that selective pressure on the capsid genes underlies temporal genetic changes in HBoV1. Notably, the major nucleotide differences distinguishing the groups were C/T substitutions at positions 4033 and 4291 of the reference sequence (Salvador 1, JQ923422), both within VP genes. These substitutions occur at wobble positions, limiting phylogenetic resolution based on VP amino acids. Unexpectedly, however, these synonymous substitutions overlap with the ORFx reading frame and alter codons at positions 25 and 111 of ORFx. Group 2 sequences encode 111I, whereas Group 1 encodes 111T, with a subset of Group 1 isolates forming a cluster defined by 25I. Thus, ORFx is the only gene that clearly distinguishes HBoV1 groups at the amino acid level. This finding suggests that ORFx may play a biological role in the viral life cycle. Although ORFx was predicted in a previous study [20], its function remains unknown, and future investigations are required to elucidate its role.

These findings also have significant implications for epidemiological monitoring. The observed year-to-year replacement of predominant HBoV1 groups provides valuable insight into the genetic dynamics of the virus, which has traditionally been considered genetically stable. Furthermore, the identification of ORFx amino acid variation as a reliable genetic marker provides a new tool that may improve genotyping accuracy and facilitate more detailed monitoring of HBoV1 transmission patterns. Although no direct association with clinical outcome was demonstrated in this study, understanding temporal genomic shifts is important for interpreting diagnostic results and anticipating potential changes in virus behaviour.

Although the number of genomes obtained each year varied, this variation reflected the availability of HBoV1-positive clinical specimens. Previous studies have shown that HBoV1 typically exhibits a single predominant genetic group within an epidemiological year, with limited within-year diversity [9,10]. Our dataset showed the same pattern: isolates collected within each year displayed very limited diversity and generally belonged to a single genetic group, even in years with only one or two sequences. Therefore, the yearly group assignment is considered reliable, and the temporal shift between Group 2 → Group 1 → Group 2 is supported by consistent within-year patterns. The continuous, region-specific surveillance from 2018 to 2024 enabled detection of these temporal genetic shifts despite annual differences in specimen availability.

This study has several limitations. Although the limited number of isolates (*n*=32) and single geographical region may restrict the generalizability of these findings, the consistent monitoring of a defined population allowed clearer detection of temporal shifts. Nearly complete genomes were examined; however, functional interpretation of the identified substitutions is limited. Although multiple nucleotide differences were detected in the VP genes, these were synonymous changes and are unlikely to affect capsid antigenicity. In contrast, ORFx – which remains uncharacterized – showed group-specific amino acid variation, raising the possibility that these substitutions may influence viral characteristics. Experimental validation of ORFx function was not performed in this study, and no association between ORFx variation and clinical features was assessed. Future studies involving larger sample sizes, multiple regions and functional assays will be essential to determine the biological and clinical relevance of ORFx and its role in HBoV1 evolution.

## Conclusion

This study demonstrates that temporal genetic changes occur among clinically circulating HBoV1 genomes and that group differentiation is determined primarily by VP genes, with ORFx providing the key amino acid-level distinction. These findings suggest a potential biological role for ORFx in the HBoV1 life cycle and highlight the need for further functional studies. This highlights the importance of continuous genomic monitoring of respiratory viruses, including HBoV1, in clinical settings.

## Supplementary material

10.1099/jmm.0.002165 Supplementary Material 1.

## Abbreviations

ALI: air–liquid interface
COVID-19: coronavirus disease 2019
HBoV1: human bocavirus 1
HBTECs: human bronchial/tracheal epithelial cells
NGS: next-generation sequencing
NP1: nuclear phosphoprotein 1
NSs: non-structural proteins
RTDT: RelTime with Dated Tips
VPs: viral capsid proteins
