# Supplementary material for: Temporal genetic changes in human bocavirus 1 in Fukushima, Japan, from 2018 to 2024
Source: J Med Microbiol. 2026 May 12;75(5):002165. doi: 10.1099/jmm.0.002165 (PMC13162154; doi:10.1099/jmm.0.002165)
Supplement: Supplementary Material 1. [file jmm-75-02165-s001.pdf]

Supplementary Figure S1

Alignment of ORFx amino acids

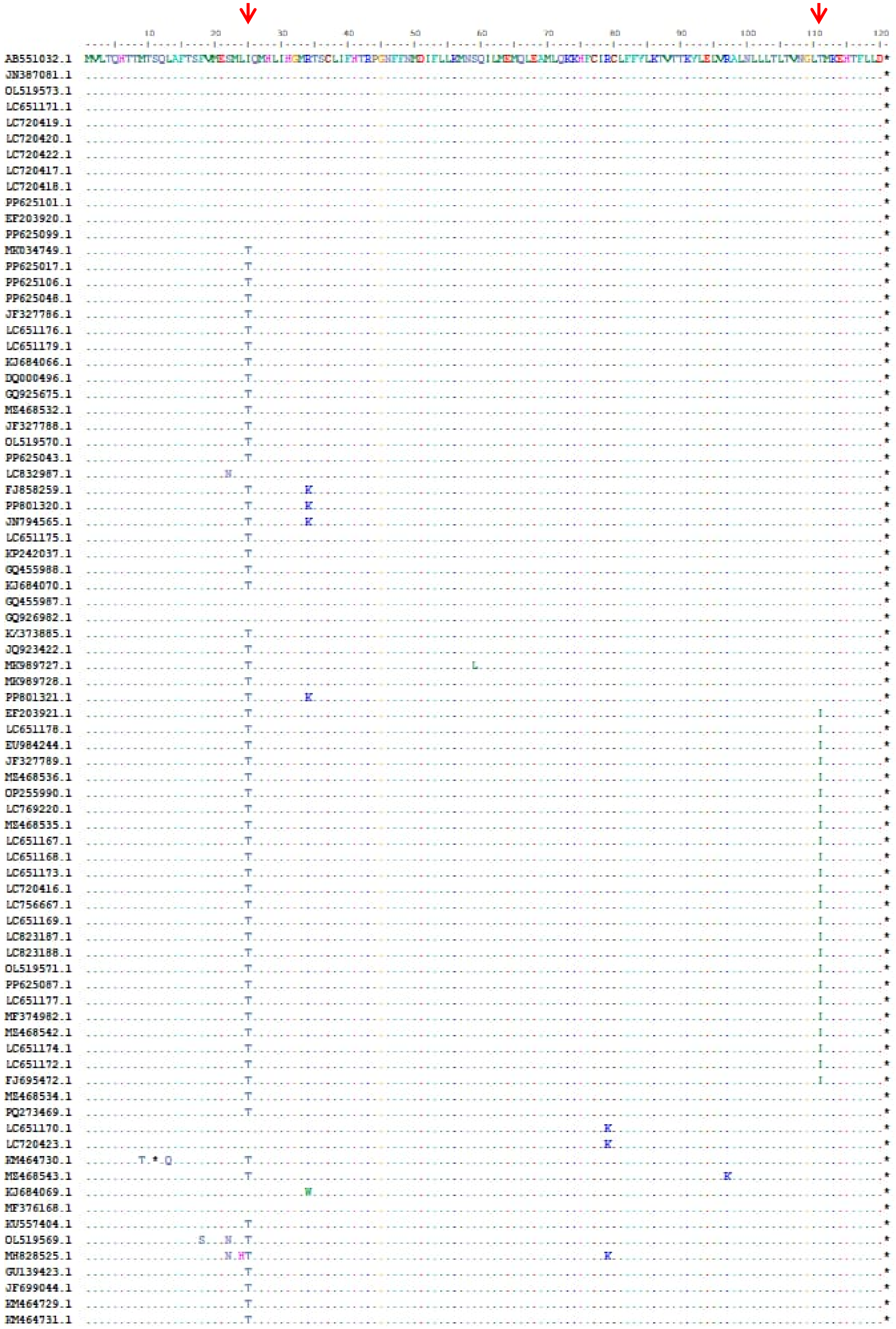

Supplementary Fig. S1. Alignment of ORFx amino acids of HBoV1.

Amino acid alignment of the ORFx region from all analysed HBoV1 genomes. Positions 25 and 111, which differ among HBoV1 groups, are indicated by arrows.
